# Excellent Cyclic and Rate Performances of SiO/C/Graphite Composites as Li-Ion Battery Anode

**DOI:** 10.3389/fchem.2020.00388

**Published:** 2020-05-15

**Authors:** Long Hu, Wenming Xia, Renheng Tang, Renzong Hu, Liuzhang Ouyang, Tai Sun, Hui Wang

**Affiliations:** ^1^Guangdong Provincial Key Laboratory of Advanced Energy Storage Materials, School of Materials Science and Engineering, South China University of Technology, Guangzhou, China; ^2^Guangdong Province Key Laboratory of Rare Earth Development and Application, Guangdong Research Institute of Rare Metals, Guangzhou, China

**Keywords:** lithium-ion battery, SiO, spray drying, pyrolysis, cyclic stability

## Abstract

The SiO-based composites containing different carbon structures were prepared from asphalt and graphite by the milling, spray drying, and pyrolysis. In the obtained near-spherical composite particles, the refined amorphous SiO plates, which are coated with an amorphous carbon layer, are aggregated with the binding of graphite sheets. The SiO/C/Graphite composites present a maximum initial charge capacity of 963 mAh g^−1^ at 100 mA g^−1^, excellent cyclic stability (~950 mAh g^−1^ over 100 cycles), and rate capability with the charge capacity of 670 mAh g^−1^ at 1,000 mA g^−1^. This significant improvement of electrochemical performances in comparison with pristine SiO or SiO/C composite is attributed to the unique microstructure, in which both the graphite sheets and amorphous carbon coating could enhance the conductivity of SiO and buffer the volume change of SiO. The higher pyrolysis temperature causes the denser spherical microstructure and better cycle life. Our work demonstrates the potential of this SiO/C/Graphite composite for high capacity anode of LIBs.

## Introduction

With the rapid development of portable electronic devices and plug-in hybrid electric vehicles, high-capacity anode materials are urgently essential to satisfy the ever-increasing energy storage density requirements of LIBs (Krywko-Cendrowska et al., 2013; Song et al., 2013; Yamamura et al., 2013; Yu et al., 2014), since the theoretical specific capacity of commercial graphite anode is limited to 372 mAh g^−1^ (Wu et al., 2014; Li et al., 2015; Wang et al., 2015; Fu et al., 2018). Silicon (Si) is one of the most promising anode materials for the next-generation Li-ion batteries because of its super-high theoretical capacity (4,200 mAh g^−1^, corresponding to Li_22_Si_5_ alloy) (Si et al., 2011; Zeilinger et al., 2013; Liang et al., 2014; Su et al., 2014; Lee et al., 2019). However, its drastic volume expansion (>300%) during the lithiation/delithiation process readily causes the pulverization and cracking of electrode and a rapid fading of cyclic capacity, hindering its commercial applications (Wang et al., 2011; Yamada et al., 2013; Rahman et al., 2016). Recently, various approaches have been adopted to develop nano-sized silicon (Kataoka et al., 2016; Jia et al., 2018), silicon-based composites (Huang et al., 2013; Yang et al., 2018), and silicon oxides (Lv et al., 2013), which could effectively overcome the pulverization problem of Si anode, and significantly improve the cyclic performance.

Silicon monoxide (SiO) also presents rather high specific capacity (2,400 mAh g^−1^) (Li et al., 2013; Wang et al., 2014). Compared with pure Si, the volume change of SiO (200%) is significantly decreased due to the introduction of oxygen. Theoretically, the cyclic capability of SiO should be more stable than Si. However, the SiO suffers from a lower intrinsic conductivity (6.7 × 10^−4^ S·cm^−1^) (Hou et al., 2015). Additionally, the reaction of SiO with Li^+^ produces much inert Li-oxide (Li_2_O) and Li-silicate (Li_4_SiO_4_) (Kim et al., 2013; Lv et al., 2015), which would lead to large initial irreversible capacity and low initial Coulombic efficiency (Kim et al., 2011; Yom et al., 2016). For that, carbonaceous materials or metals are used as useful additives to form nanocomposite structure, which could not only enhance electronic conductivity but also buffer the volume change. Thus, the electrochemical performances of SiO could be markedly improved. Wang et al. (2011) prepared the SiO/C composite by the modified Stöber method, which exhibited a capacity of 800 mAh g^−1^ after 50 cycles. Yuan et al. (2015) prepared the SiO/C composite by a hydrothermal process, which exhibited a capacity of 744 mAh g^−1^ at 0.1 C after 50 cycles. The other method is to utilize the disproportionation reaction of SiO into the Si/SiO_2_ system, within which the SiO_2_ matrix could buffer the enormous volume change of Si. For example, Hwa et al. (2013) prepared the modified SiO electrode by the disproportionation reaction, which exhibited a capacity of *ca*. 1,000 mAh g^−1^ over 50 cycles. Zhang et al. (2019) developed C-SiO-MgSiO_3_-Si secondary super-particles to address the low ICE, which exhibited the highest ICE of 78.3% with a high initial capacity of 1,608 mAh g^−1^. Xu et al. (2017) synthesized novel SiO_x_/asphalt membrane via demulsification of porous SiO_x_ microspheres. The SiO_x_/C/G anode retained a reversible capacity of 541 mAh g^−1^ after 600 cycles at 0.2 A g^−1^.

In this study, a spherical composite of amorphous SiO, amorphous carbon, and graphite was obtained by the preparation process of mechanical milling, spray drying, and heat-treatment. The obtained SiO-based composites exhibited excellent cyclic capacity. The effect of heat-treatment temperature and graphite addition on the electrochemical performances was investigated.

## Experimental

### Preparation of SiO-Based Composites

**Figure 1:**
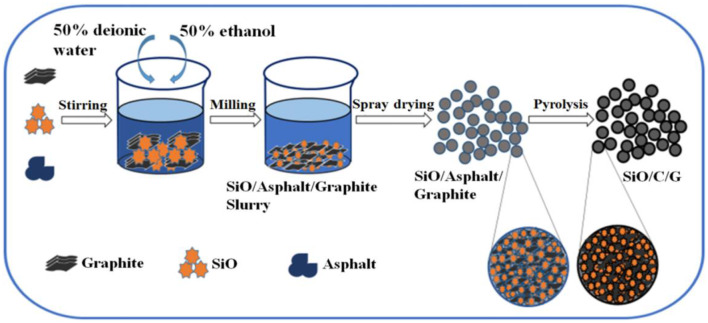
Scheme 1 Schematic representation of the synthetic process of SiO/C/G composites.

The preparing process of SiO/C/G composites is schematically shown in Figure 1. The raw materials of SiO, asphalt and graphite were mixed with the mass ratio 3:1:1 and placed in a beaker filled with 400 ml solvent containing 50% deionized water and 50% ethanol. The obtained precursor solution was mechanically milled at 2,800 rpm for 3 h, using Zirconium oxide balls of 2 mm diameter, with a ball to powder mass ratio of 20:1. After that, using a sieve to separate the slurry from balls. Then, the separated slurry was dried by spray drying under the following technological conditions: the air-feeding temperature 340°C, the air-outlet temperature 140°C, and the flowing rate 25 ml·min^−1^. Finally, the prepared precursor was heated up to 700 and 900°C at a rate of 3°C min^−1^ under nitrogen atmosphere and kept for 3 h in a tube furnace, the obtained composites heated at 700 and 900°C were termed as SiO/C/G-700 and SiO/C/G-900, respectively. For comparison, the SiO/C composite without the addition of graphite was also prepared by the same preparing process from SiO and asphalt with the mass ratio of 3:1 at the pyrolysis temperature of 700°C, the obtained composite was termed as SiO/C-700.

### Structural Characterization

The structures of the prepared composites were characterized by using X-ray diffraction (XRD, Philips X'Pert MPD, Cu Kα), scanning electron microscopy (SEM, Zeiss supra 40), Transmission electron microscope (TEM, JEOL 2100) operating at 200 kV, the Fourier Transform Infrared Reflection (FTIR, Bruker Vector 33), and laser Raman spectroscopy (HORIBA Labram) with an excitation wavelength of 632.8 nm. Thermogravimetric analysis (TG, Netzsch STA 449C) was performed to determine the carbon content and the pyrolysis temperature of the composites at a heating rate of 5 K·min^−1^ with N_2_ flowing rate of 30 ml·min^−1^.

### Electrochemical Measurement

The electrochemical performances of as-prepared composites were evaluated using 2,032 coin half-cells. The coin cells were assembled in an Ar-filled glove box. The working electrodes of tested cells were fabricated by pasting mixture slurry consisting of various active materials, acetylene black (AB) as conductive agent and carboxymethyl cellulose (CMC) as binder at a weight ratio of 60:20:20 onto a copper foil (10 cm^2^, 99.9%), the mass loading for the electrodes was 0.65–0.95 mg cm^−2^. The Li foil was used as the counter electrode and reference electrode. Porous polypropylene (Celgard 2500) was used as the separator, and the electrolyte is 1M LiPF_6_ dissolved in a mixed solvent of ethylene carbonate (EC), dimethyl carbonate (DMC) and ethyl methyl carbonate (EMC) (1:1:1 by volume). The galvanostatic cycling test is carried out with Land CTR 2001A Tester at different current densities in the voltage range of 0.01–1.5 V vs. Li/Li^+^ to measure the specific capacity, rate capability and Coulombic efficiency.

## Results and Discussion

### Microstructural Analysis

**Figure 2:**
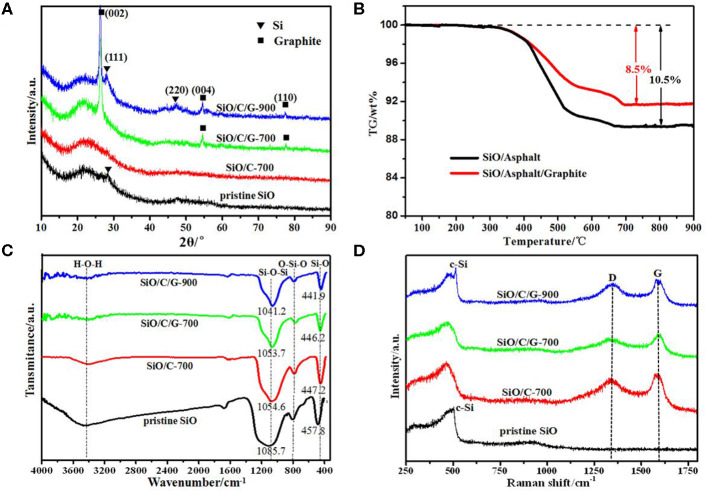
Pristine SiO, SiO/C-700, SiO/C/G-700, and SiO/C/G-900 composites material: **(A)** XRD patterns. **(B)** TG curves of SiO/Asphalt and SiO/Asphalt/G composites. **(C)** FTIR spectra. **(D)** Raman spectra.

Figure 2A shows the XRD patterns of pristine SiO and the as-prepared composites. For the pristine SiO, the broad scattering peak ranging 15–35° is assigned to amorphous SiO, and the weak Si (111) diffraction indicates that a small amount of crystalline Si retained in the amorphous SiO. The XRD pattern of SiO/C-700 composite does not show any diffraction peaks, suggesting that the asphalt transformed to amorphous carbon at the pyrolysis temperature of 700°C. Meanwhile, the retained Si in the raw SiO should be oxidized to amorphous SiO during milling. Sharp Bragg peaks of graphite are present in the XRD patterns of both SiO/C/G-700 and SiO/C/G-900 composites. Especially for the SiO/C/G-900 composite, the appearance of Si diffractions implies the partial thermal reduction reaction of SiO with carbon at 900°C, which could not occur at 700°C for the SiO/C/G-700 composite.

To determine the content of amorphous carbon in the composites, TG analysis was performed on the SiO/Asphalt and SiO/Asphalt/Graphite composites by spray drying, and the results are shown in Figure 2B. The weight loss is due to the dehydration-condensation reaction and thermal decomposition of super polymer in asphalt. Above 700°C, the TG curves remain almost unchanged, indicating the complete decomposition of asphalt. Therefore, the pyrolysis temperature of SiO/Asphalt and SiO/Asphalt/Graphite was set above 700°C. It is also shown in Figure 2B that the weight loss for the sprayed SiO/Asphalt composite (weight ratio 3:1) is 10.5 wt.%. Therefore, the content of amorphous carbon in the heated composite SiO/C-700 is calculated to be ca. 16.2 wt.% and the fraction of SiO is 83.8 wt.%. Similarly, for the SiO/C/G-700 composite, it consists of 12.6 wt.% of amorphous carbon, 21.9 wt.% of graphite and 65.5 wt.% of SiO. The SiO/C/G-900 composite should have the same composition with the SiO/C/G-700 composite, if not considering the reduction of partial SiO into Si.

Figure 2C compares the FTIR spectra of pristine SiO and the as-prepared composites. For the pristine SiO, the characteristic peaks at 1085.7 cm^−1^, 802.0 cm^−1^, and 457.8 cm^−1^ are, respectively, attributed to asymmetric Si-O-Si stretching vibration, symmetric O-Si-O deformation vibration and Si-O rocking vibration (Zheng and Li, 2014; Li et al., 2015). While for the SiO/C-700 and SiO/C/G composites, there appears obvious red shift for the Si-O-Si stretching vibration and Si-O rocking vibration. Especially for the SiO/C/G-900 composite heated at a higher temperature, it exhibits the largest red shift to 1041.2 cm^−1^ and 441.9 cm^−1^. This red shift phenomenon could be explained by the decrease of O/Si ratio in SiO_*x*_ (0 < *x* ≦ 2) as previously reported in the literature (Chang et al., 2012; Hwa et al., 2013), it also confirms the thermal reduction reaction of SiO with carbon during pyrolysis.

Figure 2D compares the Raman spectra of SiO and the as-prepared composites. For the pristine SiO, the broad bands ranging from 400 to 550 cm^−1^ are assigned to amorphous SiO (Guo et al., 2014; Rahaman et al., 2016), while a sharp weak peak at *ca*. 520 cm^−1^ corresponds to crystalline Si (c-Si). The Si peak disappears in the Raman spectra of SiO/C-700 and SiO/C/G-700 composites but appears again for the SiO/C/G-900 composite. This is well consistent with the XRD results as shown in Figure 2B. Compared with pure SiO, there is no distinct change of amorphous SiO peak in the Raman spectra of three composites. Concerning the typical D-band at 1,345 cm^−1^ and G-band at 1,585 cm^−1^ in the Raman spectrum of SiO/C-700 composite, which confirms the transformation of asphalt into disordered carbon. Besides, the Raman spectra of SiO/C/G-700 and SiO/C/G-900 composites show similar D-band and G-band peaks with that of SiO/C-700 composite, despite the addition of graphite (Xiao et al., 2013; Vengudusamy et al., 2014).

**Figure 3:**
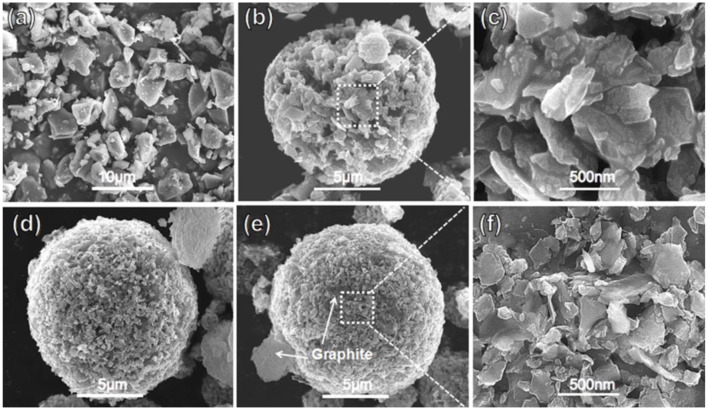
SEM images of **(a)** Pristine SiO. **(b,c)** SiO/C-700 composite. **(d)** SiO/C/G-700 composite. **(e,f)** SiO/C/G-900 composite.

Figure 3 shows the SEM images of pristine SiO and as-prepared composites. The SEM observation from Figure 3a reveals the plate-like shape of pristine SiO, which is in micrometer size. For the SiO/C-700 composite shown in Figures 3b,c, the spherical-like particles of *ca*. 15 μm in diameter are observed, within which the SiO plates of ca. 500 nm size are loosely aggregated. Therefore, the milling greatly refined the size of SiO plates. As also shown in Figures 3c,f, it appears that the SiO plates are coated by pyrolytic amorphous carbon. As seen in Figures 3d,e, both the SiO/C/G-700 and SiO/C/G-900 composites present near-spherical particles, in which the graphite sheets are dispersive to fill the pores between SiO plates. The pores of SiO/C/G-900 composites are finer because the disproportionation and thermal reduction reactions are more complete at higher pyrolysis temperature. Therefore, the SiO/C/G-900 composite particles are denser than the SiO/C-700 composite.

**Figure 4:**
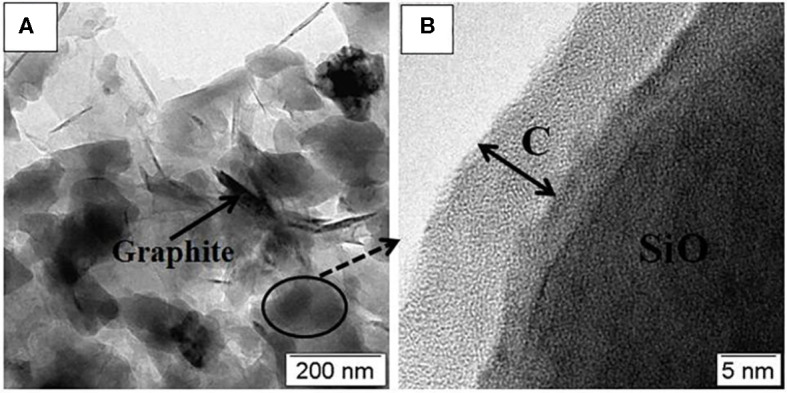
**(A,B)** TEM images of the SiO/C/G-900 composite.

The microstructure of SiO/C/G-900 composite is further characterized by TEM. Figure 4A shows the SiO is evenly distributed over the graphite sheets. As seen in Figure 4B, the SiO is wrapped with amorphous carbon layer of the thickness of ~10 nm. Overall, the multi-phase microstructure containing amorphous SiO plates and different carbon structures is formed in the SiO/C/G composites.

### Electrochemical Performances

**Figure 5:**
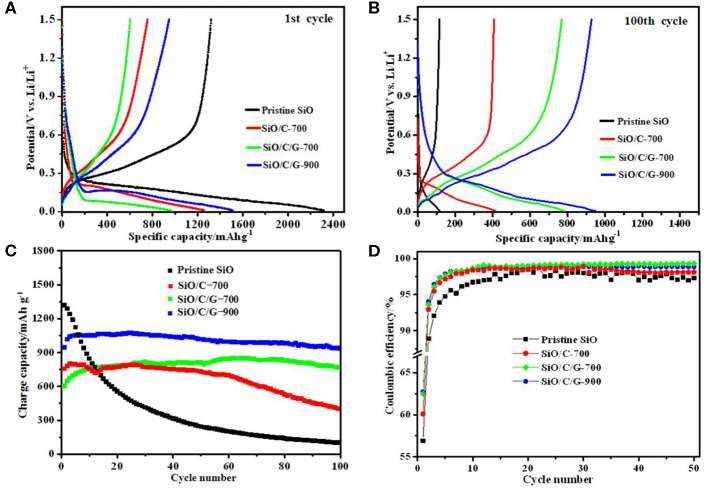
Comparison of electrochemical performances of pristine SiO, SiO/C-700, SiO/C/G-700, and SiO/C/G-900 composites at 100 mA·g^−1^: **(A,B)** The discharge-charge profiles at 1st and100th cycle. **(C)** cyclic capacity. **(D)** cyclic Coulombic efficiency.

Figure 5 compares the charge/discharge profiles, cyclic capacity, and cyclic Coulombic efficiency of pristine SiO and as-prepared composites. As shown in Figure 5A, the pristine SiO anode delivers an initial discharge capacity of 2,318 mAh g^−1^, which is slightly less than its theoretical capacity of ca. 2,400 mAh g^−1^. The initial charge capacity of pristine SiO is only 1,285 mAh g^−1^, with the initial Coulombic efficiency is 55.4%. This irreversible capacity loss is mainly due to the generation of inactive Li_2_O and Li_4_SiO_4_ in the initial discharging process. Comparatively, three composites exhibit lowered initial discharge capacity, being 1,259 mAh g^−1^ for the SiO/C-700 composite, 965 mAh g^−1^ for the SiO/C/G-700 composite, and 1,509 mAh g^−1^ for the SiO/C/G-900 composite. The much higher capacity for the SiO/C/G-900 composite than the SiO/C/G-700 composite is related to the formation of a small amount of Si, and the decrease of oxygen concentration at a pyrolysis temperature of 900°C, which has been proven in Figures 2A,C. As also shown in Figure 5A, the lowered discharge potential of three composites in comparison with pristine SiO should be due to the amorphous carbon coated on the SiO plates, which is unbeneficial to the Li^+^ diffusion to react with SiO. As shown in Figure 5B, after 100 charge-discharge cycles, the SiO/C/G-700 and SiO/C/G-900 composites possess elevated discharge potential, which is a little higher than that of the SiO/C-700 composite.

From Figure 5C, both the SiO/C/G-900 and SiO/C/G-700 composites show stable cyclic performances. The SiO/C/G-900 composite presents an initial charge capacity of 963 mAh g^−1^, which increases up to maximum 1,073 mAh g^−1^ at 25th cycle, and then slowly decreases to 950 mAh g^−1^ after 100 cycles. Therefore, the capacity retention relative to the first charge capacity is 98.7% at 100th cycle for the SiO/C/G-900 composite, which is much higher than those for pristine SiO (4.3%) and SiO/G-700 composite (52.7%). Xia et al. (2019) improve the electrochemical performance of SiO@C/graphite shows a high capacity retention rate of over 91% after 100 cycles, which is lower than the SiO/C/G-900 composite. For the SiO/C/G-700 composite, the 100th charge capacity (768 mAh g^−1^) is even much higher than its initial capacity of 603 mAh g^−1^, while for the SiO/C-700 composite, the capacity shows fast fading after 60 cycles. Therefore, the graphite addition greatly improves the cycle stability of SiO plates with amorphous carbon coating.

From Figure 5D, three composites show a little improvement in the cyclic Coulombic efficiency in comparison with pristine SiO. The highest initial Coulombic efficiency is 63.8% for the SiO/C/G-900 composite. After several cycles, the Coulombic efficiency of SiO/C/G-900 and SiO/C/G-700 composites are stabilized at 98.7%, slightly higher than those of pristine SiO and SiO/C-700 composite. The poor initial Coulombic efficiency originating from its low electrical conductivity and irreversible reaction during the first cycle (Choi et al., 2019).

**Figure 6:**
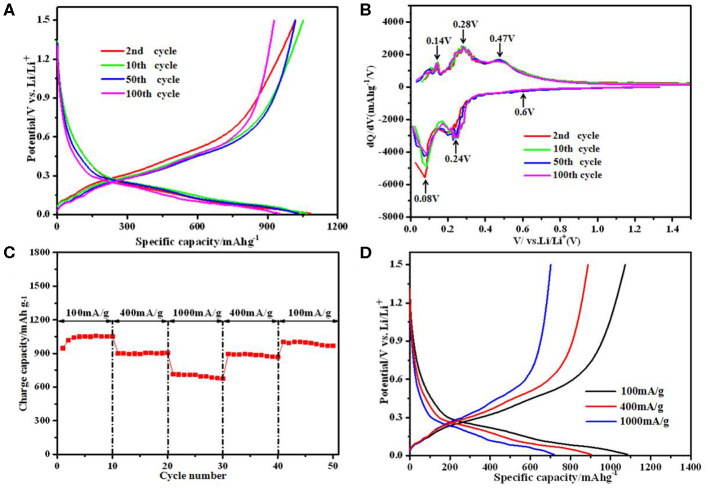
Electrochemical performance of the SiO/C/G-900 composite: **(A)** The discharge-charge profiles. **(B)** differential capacity plots **(C)** rate capability. **(D)** discharge-charge profiles at different current densities.

Since the SiO/C/G-900 composite shows the best electrochemical performances, Figure 6A further shows the selected cyclic charge/discharge profiles of SiO/C/G-900 composite. There is no obvious change for the charge-discharge profiles at 2nd, 5th, 50th, 100th cycles, indicating the good reversibility of the reactions in this composite anode. To investigate the lithiation/delithiation mechanism of the SiO/C/G-900 composite, the differential capacity plots at different cycles are depicted in Figure 6B. A weak cathodic peak at 0.60 V in the 1st cycle is ascribed to the formation of SEI film and decomposition of electrolyte, contributing some irreversible capacity (Zhou et al., 2013; Hubaud et al., 2015). Besides, the reduction peak at 0.24 V is attributed to the alloying of Si with Li^+^, which corresponds to two anodic peaks at 0.28 V and 0.47 V, reflecting a two-step dealloying process (Tao et al., 2012; Xie et al., 2014). A pair of cathodic peak at 0.08 V and anodic peak at 0.14 V belongs to the lithium insertion and extraction process of graphite (Zhou et al., 2013; Hang et al., 2014). The overlapping peaks of the cyclic dQ/dV profiles also indicate excellent cyclic stability for the SiO/C/G-900 composite.

Figure 6C shows the good rate capability of the SiO/C/G-900 composite. At 400 mA g^−1^, the composite exhibits stable reversible charge capacity of ca. 895 mAh g^−1^, which is a little lower than maximum charge capacity of 1,017 mAh g^−1^ at 100 mA g^−1^. The SiO_x_@C electrode exhibits rate capability with discharge capacity of approximately 946.7, 751.7 at 0.2, and 0.4 A/g (Cui et al., 2016), which is lower than SiO/C/G-900 composite. Even at 1,000 mA g^−1^, a charge capacity of 670 mAh g^−1^ is maintained after 10 cycles. When the current density is back to 100 mA g^−1^ after 40 cycles, the charge capacity recovers to 980 mAh g^−1^. Combined with the corresponding charge/discharge profiles (Figure 6D) at different current densities, the SiO/C/G-900 composite shows excellent rate capability.

As abovementioned, the SiO/C/G-900 composite heated at 900°C shows high capacity, excellent cyclic stability, and rate capability. The higher pyrolysis temperature for the SiO/C/G-900 composite than the SiO/C/G-700 composite results in the formation of a small amount of Si crystals in the amorphous SiO matrix, this is responsible for the increased capacity. Among three composites, the excellent cyclic stability of the SiO/C/G-900 and SiO/C/G-700 composites is mainly attributed to graphite sheets, which helps to form dense microstructure in the near-spherical composite particles. In contrast, the loose microstructure for the SiO/C-700 composite leads to fast capacity fading after tens of cycles. Therefore, this combination of amorphous carbon coating and graphite sheets could effectively buffer the volume change of SiO during repeatedly lithium insertion/extraction, and maintain the structural stability of the composite electrode. Previous literature reported the improvement of cyclic performances of SiO by compositing with different carbon materials (Cui et al., 2016; Xia et al., 2019). Comparatively, this work shows that better cyclic performances could be achieved by compositing hybrid carbon structure with SiO.

## Conclusions

We demonstrate a facile route to prepare high-performance SiO-based composite anode. By mechanical milling, spray drying, and high-temperature pyrolysis, the obtained SiO/C/G-900 composite exhibited an initial charge capacity (~963 mAh g^−1^ at 100 mA g^−1^), excellent cyclic stability (~950 mAh g^−1^ over 100 cycles) and rate capability. The enhanced electrochemical performances are attributed to the unique composite microstructure of SiO/C/G composites, in which both amorphous carbon coating and the graphite sheets improve the conductivity as well as the stability of composite electrode during repeatedly charge-discharge process. Therefore, our work shows that this SiO/C/G composite is a promising candidate for the anode material of next-generation LIBs.

## Data Availability Statement

The datasets generated for this study are included in this article.

## Author Contributions

HW, RH, and RT designed the experiments and finished the writing of the manuscript. LH and WX completed the experiments and analyzed the data. LO and TS provided helpful suggestions on the preparation process of electrode materials. All authors have made a substantial and direct contribution to this work.

## Conflict of Interest

The authors declare that the research was conducted in the absence of any commercial or financial relationships that could be construed as a potential conflict of interest.
